# Association of Enterovirus D68 with Acute Flaccid Myelitis, Philadelphia, Pennsylvania, USA, 2009–2018

**DOI:** 10.3201/eid2509.190468

**Published:** 2019-09

**Authors:** Priyanka Uprety, Darcy Curtis, Michael Elkan, Jeffrey Fink, Ramakrishnan Rajagopalan, Chunyu Zhao, Kyle Bittinger, Stephanie Mitchell, Erlinda R. Ulloa, Sarah Hopkins, Erin H. Graf

**Affiliations:** Children’s Hospital of Philadelphia, Philadelphia, Pennsylvania, USA (P. Uprety, D. Curtis, M. Elkan, J. Fink, R. Rajagopalan, C. Zhao, K. Bittinger, E.R. Ulloa, S. Hopkins, E.H. Graf);; University of Pennsylvania, Philadelphia (P. Uprety, K. Bittinger, S. Mitchell, E.R. Ulloa, S. Hopkins, E.H. Graf)

**Keywords:** enterovirus, enterovirus D68, acute flaccid myelitis, Pennsylvania, Philadelphia, children, viruses, whole-genome sequencing, United States, EV-D68

## Abstract

Acute flaccid myelitis (AFM) is a polio-like disease that results in paralysis in previously healthy persons. Although the definitive cause of AFM remains unconfirmed, enterovirus D68 (EV-D68) is suspected based on 2014 data demonstrating an increase in AFM cases concomitant with an EV-D68 outbreak. We examined the prevalence in children and the molecular evolution of EV-D68 for 2009–2018 in Philadelphia, Pennsylvania, USA. We detected widespread EV-D68 circulation in 2009, rare detections in 2010 and 2011, and then biennial circulation, only in even years, during 2012–2018. Prevalence of EV-D68 significantly correlated with AFM cases during this period. Finally, whole-genome sequencing revealed early detection of the B1 clade in 2009 and continued evolution of the B3 clade from 2016 to 2018. These data reinforce the need to improve surveillance programs for nonpolio enterovirus to identify possible AFM triggers and predict disease prevalence to better prepare for future outbreaks.

A large outbreak of enterovirus D68 (EV-D68) in children in the United States caught national attention during late summer and fall of 2014. That outbreak was characterized by severe lower respiratory tract disease resulting in respiratory failure in many cases (*1*); a paralytic condition occurred in a smaller subset of children (*2*). Previous reports of invasive EV-D68 disease had been described across the globe (*3*,*4*), but the magnitude of this 2014 outbreak with regard to childhood illness was unprecedented. The Centers for Disease Control and Prevention (CDC) called for national heightened awareness, particularly for rapid onset of a traumatic limb weakness along with radiologic evidence of gray matter lesions, coined acute flaccid myelitis (AFM) (*2*,*5*–*7*). A second period of EV-D68 circulation, again associated with severe respiratory disease, was described during late summer and fall of 2016, although 2 studies suggest lower levels of circulation during that period than in 2014 (*8*,*9*). Despite a documented spike in AFM in US children during fall of 2016 and 2018 (*10*), EV-D68 surveillance data from this time are limited (*11*,*12*). In addition, little is known about the circulation of EV-D68 and association with AFM before 2014.

Although the definitive cause of AFM remains unknown, growing evidence (*13*,*14*) supports the association between EV-D68 and AFM. Epidemiologists have found that similar temporal associations between the 2014 EV-D68 outbreak and AFM cases also occurred in other countries (*14*). Hixon et al. found that paralysis could be elicited in mice infected with EV-D68 strains from the 2014 outbreak (*15*). As in children with AFM, these EV-D68–infected mice had a loss of motor neurons in the anterior horns of spinal cord segments corresponding to paralyzed limbs. Last, virus isolated from spinal cords of infected mice transmitted disease when injected into naive mice (*15*). 

Despite the mounting evidence (*13*,*14*), the association between EV-D68 and AFM remains controversial. The detection of EV-D68 primarily in respiratory specimens with a concomitant lack of regular detection in cerebrospinal fluid (CSF) (*7*,*16*) continues to cast doubt, even though other neurotropic viruses (such as polioviruses) have similar detection patterns (*14*).

What remains to be shown for EV-D68 is whether its genome has changed over time to enable increased neurotropism. Since the first description of the nonneuroinvasive EV-D68 prototype in 1962, different genetic clades associated with neuroinvasion have been identified (*15*). Whole-genome sequencing (WGS) of the 2014 epidemic strains (dominated by clade B1) demonstrated a possible association between specific polymorphisms in EV-D68 and their overlap with other neurotropic and AFM-causing enteroviruses, such as poliovirus or enteroviruses D70 and A71 (*16*). These data suggest that the EV-D68 genome has changed over time to enable neurotropism or possibly increased virulence resulting in more widespread disease. Given that most studies have been based on partial instead of WGS, we also might be underestimating the number of clinically important genetic mutations in EV-D68 that drive increased infections with poor patient outcomes. Recognizing that longitudinal analyses of viral patterns and the molecular evolution of EV-D68 by WGS are sparse, we investigated the prevalence of EV-D68 in children in the Children’s Hospital of Philadelphia (CHOP; Philadelphia, PA, USA) during 2009–2018 in relationship to AFM cases. We also investigated the phylogenetic relationships and genotypic features through WGS during this period to shed light on relevant mutations from year to year.

## Materials and Methods

### Specimen Selection

Nasopharyngeal aspirate samples that tested positive for rhinovirus/enterovirus (RV/EV) by laboratory-developed real-time reverse transcription PCR (rRT-PCR) through routine clinical diagnostic testing at CHOP were included in this study. Specimens have been archived and stored in multiple aliquots at −80°C since 2009. Based on data from prior publications (*8*,*9*), we selected a subset (every 5th specimen) of RV/EV-positive samples from August 1 through November 30 of 2009–2018 for subsequent testing for EV-D68. In total, we tested 1,433 specimens across the 10-year period for EV-D68. We further subjected a smaller subset of those positive for EV-D68 (26 samples) and residual EV-D68–positive cases of AFM (4 samples) to shotgun metagenomic RNA sequencing. In addition to these specimens, we also tested residual RV/EV-positive nasopharyngeal aspirates from 12 children confirmed to have AFM for EV-D68 by rRT-PCR. Detailed methods are provided in the Appendix). The Institutional Review Board of CHOP approved this study (IRB 16-012987). 

### AFM Cases

We retrospectively identified AFM cases from 2009 to initiation of CDC surveillance in 2014 through a search of International Classification of Diseases, 9th Revision, diagnosis codes in CHOP’s electronic health record system and a text search of spinal magnetic resonance imaging (MRI) reports (M.M. Cortese, CDC, pers. comm. 2019 Jan 14). A neurologist reviewed MRI images and clinical information to identify cases meeting the 2014 CDC criteria for confirmed AFM. We identified cases from 2014 on prospectively. AFM cases were considered confirmed if they met the CDC criteria of illness with the acute onset of flaccid limb weakness and MRI demonstrating lesions restricted to the gray matter of the spinal cord that span >1 vertebral segments.

### Statistical Analyses

All statistical analyses were performed in GraphPad Prism version 7.0 (GraphPad Software, San Diego, CA, USA). These analyses comprised Spearman correlation and linear regression analyses.

## Results

### Prevalence of EV-D68 and Association with AFM

**Figure 1:**
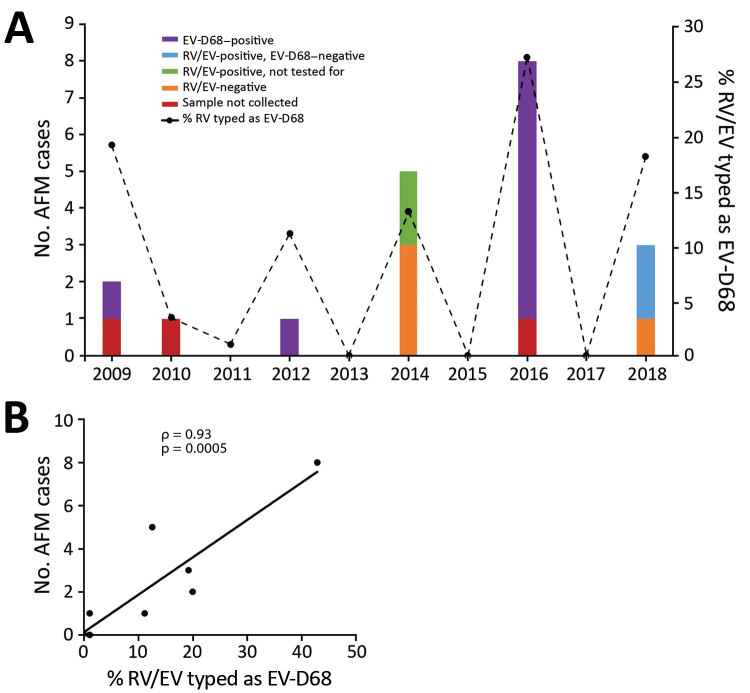
Association between AFM and EV-D68 prevalence, Philadelphia, Pennsylvania, USA, 2009–2018. A) The percentage of nasopharyngeal aspirate samples positive for RV/EV that typed as EV-D68 by real-time reverse transcription PCR. B) Comparison of confirmed AFM cases with the prevalence of EV-D68 during the same time period using Spearman correlation ρ and linear regression analysis (n = 10). AFM, acute flaccid myelitis; EV-D68, enterovirus D68; RV/EV, rhinovirus/enterovirus.

We tested 1,433 respiratory specimens that were PCR-positive for RV/EV from August 1 through November 30 of 2009–2018 for EV-D68 by rRT-PCR. EV-D68 was detected in all years except 2013, 2015, and 2017 (Figure 1). Detections in 2010 and 2011 were rare—3 samples were positive in 2010 and 1 in 2011—and <4% of all RV/EV-positive samples tested those years were typed as EV-D68 (Figure 1, panel A). In contrast, >10% of RV/EV-positive specimens typed as EV-D68 in 2009, 2012, 2014, 2016, and 2018. The highest percentage occurred in 2016, when 27% of RV/EV-positive specimens (30/112 RV/EV-positive samples tested) were typed as EV-D68. In addition, we observed a 19% (35/180 RV/EV-positive samples tested) prevalence of EV-D68 in 2009 and an 11% (12/107 RV/EV-positive samples tested) prevalence of EV-D68 in 2012. The circulation appears to follow a biennial pattern from 2012 to 2018; however, a 2-year gap occurred in widespread circulation during 2009–2012, breaking this every-other-year cycle.

AFM cases from CHOP identified during the same 10-year period showed a similar biennial pattern of presence/absence during 2012–2018 (Figure 1, panel A); the highest number were identified in 2016. Two AFM cases were identified in 2009 when EV-D68 circulation was high and 1 in 2010 when circulation was low among the study population, and no cases were identified in 2011. Because the association between EV-D68 and AFM and subsequent importance of respiratory specimen collection were not recognized until late 2014, respiratory samples were not routinely collected for investigation of AFM at CHOP before 2016. During 2009–2018, a total of 20 AFM cases were identified. For 17 of these, respiratory specimens were collected at the time the child was brought to the hospital for care, and 14 (82%) of respiratory specimens tested positive for RV/EV (Figure 1, panel A). Twelve RV/EV-positive respiratory specimens taken from children in whom AFM was diagnosed had a residual sample for retrospective EV-D68 testing. Ten (83%) of these tested positive for EV-D68, including 7 from 2016 (Figure 1, panel A). One of the children who tested positive, from 2009, had a respiratory sample collected that tested positive for RV/EV, but a residual sample was not available for retrospective analysis. However, a specimen collected 21 days later that also tested positive for RV/EV was available for retrospective EV-D68 typing. This sample was positive for RV/EV at a crossing cycle threshold (C_t_) of 42 and typed as EV-D68 at a C_t_ of 38. The specimen collected when care was sought had a C_t_ of 22 for RV/EV, suggesting the sample from 21 days later was not a new infection. The remaining 10 AFM children who were not tested for EV-D68, or were negative, had either no infectious etiology identified (7 cases), an RV/EV detected that was not typed (2 cases), or a non-D68 enterovirus identified (1 case).

The high prevalence of EV-D68 in 2016 coincided with a high number of confirmed AFM cases at CHOP and led us to question whether the association could be investigated further back. We compared the number of AFM cases identified from August through November during 2009–2018 with the percentage of RV/EV-positive respiratory samples that typed as EV-D68 during the same period. We found a significant positive linear correlation between these 2 values over the decade sampled (Spearman ρ 0.93; p = 0.0005; Figure 1, panel B). This finding suggests a strong association between circulation of EV-D68 and increases in AFM in children at CHOP.

### Phylogenetic Analysis of EV-D68 WGS

To determine which clades were circulating in CHOP, we performed metagenomic sequencing on a subset of EV-D68–positive specimens collected during 2009–2018 from children without AFM (26 specimens) and from children with AFM (4 cases) for whom a residual sample was available. We were able to assemble complete polyprotein sequences from 28 of the 30 samples; 1 sample from 2009 and 1 from 2018 did not have sufficient EV-D68 genome coverage and were excluded from the analysis.

**Figure 2:**
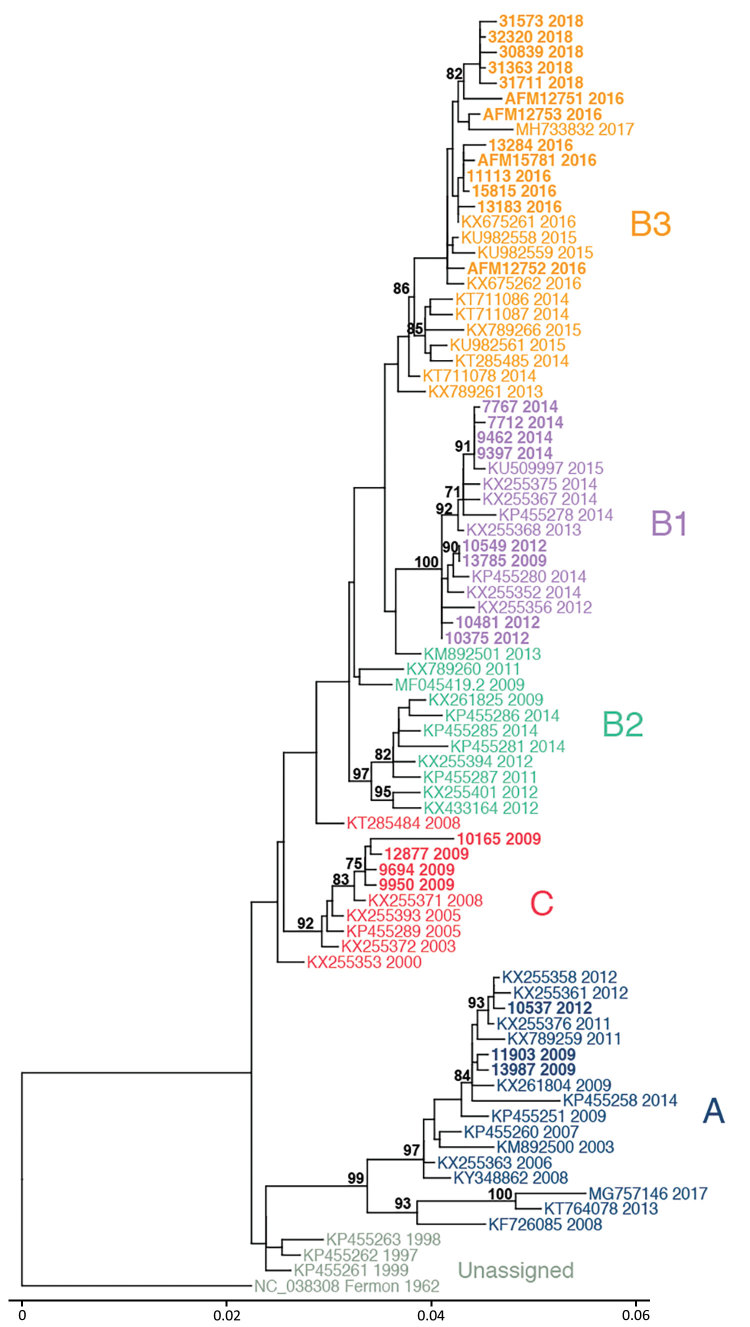
Phylogenetic relationships between Philadelphia whole enterovirus D68 (EV-D68) genomes, including AFM cases, Philadelphia, Pennsylvania, USA, 2009–2018. EV-D68 polyprotein sequences from GenBank (n = 55) and this study (n = 28) were used to build a maximum-likelihood tree. EV-D68 clades, defined in prior publications, are indicated. Sequences from this study are indicated in bold, and AFM cases are indicated with that prefix. AFM, acute flaccid myelitis. Scale bar indicates nucleotide substitutions per site.

Phylogenetic analysis showed that EV-D68 from the study population in 2009 belonged to clade A (2 samples) and clade C (4 samples); 1 sample belonged to clade B1 (Figure 2, no. 13785). The clade B1 sample from 2009 showed 99.8% pairwise identity with a clade B1 sample from 2012 (Figure 2, no. 10549). By 2012, clade B1 was more common (3 samples); 1 sample belonged to clade A. As expected in 2014, all samples clustered in clade B1 but at a distinctly different lineage from the 2012 viruses identified from the study population (Figure 2). The 2016 samples clustered in clade B3, consistent with previous observations (*17*). AFM cases were interspersed with non-AFM cases from the same period. Phylogenetic analysis indicates that the 2016 clade B3 virus emerged from a common ancestor shared between the 2012 and 2014 clade B1 viruses and did not evolve directly from a previously circulating virus. In 2018, the samples continued to cluster in the B3 clade but formed a lineage distinct from the 2016 samples.

### Protein and Nucleotide Changes in the EV-D68 Genome Circulating in Philadelphia during 2009–2018

We compared amino acid (aa) substitutions across the entire 2,190-aa polyprotein among the 28 whole EV-D68 genome sequences from our study. We observed 46 aa substitutions when we compared sequences across all years (Appendix Figure, panel A), many of which were previously described when comparing 2014 clade B1 and 2016 clade B3 viruses circulating in New York state (Appendix Table 1) (*17*). Not surprisingly, most of these (25 aa substitutions) were present in the viral capsid proteins, which are the primary focus of host immune responses but are also responsible for receptor binding and cell tropism (*18*). Only 2 aa substitutions, both in the viral protein (VP) 1 protein, were different for most viruses circulating in Philadelphia in 2016 and 2018. All other amino acid positions are largely conserved between 2016 and 2018 viruses. In a prior publication, 6 aa residues were implicated for possible neurotropism because of conservation between other neurotropic enteroviruses with different identities from nonneurotropic enteroviruses as well as rhinoviruses (*16*). These 6 residues (Appendix Table 1) were present in circulating 2014 viruses in our study. However, in 2016 and 2018, including the 4 AFM cases from CHOP that we were able to subject to WGS from 2016, the amino acid in these 6 positions were the same as the dominant sequence in 2009. We did not identify any conserved aa substitutions between sequences from the 2016 AFM cases that were not also conserved in other 2016 sequences from the CHOP population.

To investigate differences that might explain changes in neurotropism, we compared aa substitutions between the nonneurotropic Fermon strain and the CHOP sequences. We found 37 aa substitutions between the prototype Fermon strain and all circulating strains in Philadelphia during 2009–2018 (Appendix Table 1; Appendix Figure 1, panel A, bottom row). Twenty-six (70%) of these substitutions were present in the structural region of the polyprotein, VP4–VP1. To further investigate the potential significance of these 37 substitutions, we analyzed the changes through scoring as described by Grantham (*19*). A substitution at position 1641, different from the 1962 strain but conserved among all strains circulating in Philadelphia during 2009–2018, appears to have the highest deleterious effect on the virus (Appendix Figure 1, panel B). This change, from cysteine to tyrosine, falls in the 3C viral protease that is responsible for cleaving the viral polyprotein. Other mutations with high scores include positions 379, 391, and 849, which correspond to the VP3 (379 and 391) and VP1 (849) capsid regions, respectively.

Like the structural proteins, the 5′ untranslated region of enteroviruses is also implicated in cell tropism and neuropathogenesis because of differences in translational efficiency (*20*). We saw little diversity in this region between years with 8 single-nucleotide polymorphisms between years and 1 nt (620) that was deleted in all 2018 sequences but was not in prior years (Appendix Table 2). A previously identified deletion that has been described in sequences from the United Kingdom and Japan (*21*) was also observed in all 2014–2018 sequences and was found in 4 of 7 sequences from 2009 and in 3 of 4 from 2012. As with the amino acid comparisons, we found no genetic changes in the 5′ untranslated region of all 4 AFM cases that were not also conserved in other non-AFM sequences from the same year. We identified 2 differences between the prototype 1962 Fermon strain and all EV-D68 in our study. A single deletion at nt 29 was observed in the Fermon strain, as well as a large deletion from nt 682–704 (Appendix Table 2) that previously was described in the same study from the United Kingdom and Japan (*21*).

## Discussion

In this 10-year longitudinal study, we showed that EV-D68 circulated in a biennial pattern among children at a hospital in Philadelphia, and circulation was widespread in even years, except for a peak in 2009 and largely absent circulation in 2010–2011. AFM cases peaked during the same years in the study population, and prevalence of EV-D68 correlated significantly with the number of AFM cases during the past decade. Half of the AFM cases were associated with EV-D68 through retrospective detection in archived respiratory samples, including a case from 2009 that would be the second earliest detection of an association between EV-D68 and AFM (*22*). For 10 EV-D68–positive cases, no other infectious etiology was identified despite extensive testing.

Genomic analysis revealed an interesting evolutionary pattern of EV-D68 in the CHOP population. As we reported in this study, clades A and C dominated in 2009. However, we also identified a single specimen with clade B1 sequence, which slightly predates modeled estimates (*16*). The evolution of lineages within a clade during 2012–2014 and again during 2016–2018 sharply contrasted with the introduction of the new B3 clade from 2014 through 2016. The 2016 clade B3 virus emerged from a common ancestor of the 2012–2014 clade B1 viruses rather than directly evolving from them. It is possible this happened over time from a minor population in Philadelphia. However, it is more likely that a new EV-D68 was introduced from a location outside the region, given that the B3 clade had been reported in 2015 (*23*), predating the 2016 CHOP outbreak. This finding has important implications about immunity within Philadelphia because we might expect another clade to emerge in 2020 based on this pattern.

US and worldwide circulation patterns of EV-D68 remain unclear. Like other enteroviruses, EV-D68 appears to skip some years in certain locations, although the years skipped are not universal across continents, even in the same hemisphere. For example, despite a high prevalence of EV-D68 even among adults in Europe in 2010 (*3*,*4*,*21*,*24*) and in Thailand in 2010 and 2011 (*25*), it was nearly quiescent in the United States during that time. Surveillance data from CDC indicate EV-D68 was the most commonly detected enterovirus in 2009, with less frequent detection in 2011 and almost complete absence in 2010 and 2012 (*26*). The peaks in 2012, 2014, and 2018 were consistent with reports from other US pediatric institutions (*11*,*12*). However, the increase we found in 2016, while consistent with reports from the Netherlands (*27*), was greater than that reported in 2 other studies of US children (*8*,*9*). Taken together, these data suggest that local pockets of transmission can occur, leading to bias in surveillance programs that rely on passive reporting or submission of specimens.

Despite mounting data, EV-D68–associated AFM remains controversial given that EV-D68 is rarely isolated from CSF. Only a handful of cases have identified EV-D68 in the central nervous system, including 1 postmortem detection (*13*,*22*). In our study, we focused on respiratory specimens because the respiratory tract is the most common site of EV-D68 replication. Consistent with other studies (*16*), enterovirus was not detected by standard-of-care PCR in the CSF in the cohort of EV-D68-positive children with AFM at CHOP. That being said, the cases of AFM positive for EV-D68 we describe had no other infectious etiology identified despite extensive testing.

Our study had several limitations, including small sample size and failure to detect an infectious etiology in 9 AFM cases. Of these 9 children, 7 had no positive infectious testing (although 3 did not have a respiratory specimen collected/tested) and 2 had a respiratory specimen positive for RV/EV, but the type was not identified. It remains possible that these children were sampled too late, because earlier respiratory sampling (<1 week from symptom onset) is associated with higher detection rates of EV-D68 (*7*).

Our study has several other important limitations. Archived specimens were tested after storage in −80°C for up to almost a decade. Although it is unlikely that viral RNA degraded during this time because these samples were not thawed before use for this study, it remains possible that EV-D68 detection rates could have been higher in prospectively tested specimens. With the exception of AFM samples, there was selection bias to sequence specimens with higher viral titers ensuring genome coverage. As a result, the clades observed might be misrepresentations for each year. Although samples with weaker viral titers are unlikely to track with specific clades, it is possible. Finally, all of the samples in this study were collected from a tertiary-care hospital serving a limited catchment area of 1 state, making it difficult to extrapolate EV-D68 circulation patterns and strain diversity over broader geographic regions of the United States.

The data presented here strengthen the association between EV-D68 infection and AFM. They also support the need for continued surveillance for EV-D68 in the United States and worldwide as the virus continues to circulate perhaps biennially, with unique prevalence in geographic pockets. Genomic analysis, particularly WGS, is also critical to clarify transmission, clade changes, and subsequent implications for immunity. WGS is also important for the association of specific genomic changes with the ability of the virus to cause AFM, requiring experimental analysis of mutations analogous to the extensive work done in poliovirus and enterovirus 71. Overall, these data reinforce the need to improve EV-D68 surveillance programs as a means of identifying possible AFM triggers that may lead to therapeutic interventions for this severe condition.

Appendix Additional methods and results for study of the association of enterovirus D68 with acute flaccid myelitis, Philadelphia, Pennsylvania, USA, 2009–2018.
