# Supplementary material for: Association of Enterovirus D68 with Acute Flaccid Myelitis, Philadelphia, Pennsylvania, USA, 2009–2018
Source: Emerg Infect Dis. 2019 Sep;25(9):1676–82. doi: 10.3201/eid2509.190468 (PMC6711208; doi:10.3201/eid2509.190468)
Supplement: Appendix — Additional methods and results for study of the association of enterovirus D68 with acute flaccid myelitis, Philadelphia, Pennsylvania, USA, 2009–2018. [file 19-0468-Techapp-s1.pdf]

Article DOI: https://doi.org/10.3201/eid2509.190468

Association of Enterovirus D68 with Acute Flaccid Myelitis, Philadelphia, Pennsylvania, USA, 2009–2018

Appendix

Methods

Real-Time Reverse Transcription PCR

The RV/EV assay was validated and approved for clinical use at CHOP in 2009 using previously published primers and probes targeting the 5′ untranslated region common to all enteroviruses and rhinoviruses (*1*). A targeted rRT-PCR for EVD68, using primers and probes with specific sequence match for EVD68 in the VP1 region, was modified from a prior publication (*2*). For all rRT-PCR assays, total nucleic acid was extracted from 200 µL of nasopharyngeal aspirates using a MagNA Pure total NA kit on the MagNA Pure automated extractor (Roche, Indianapolis, IN, USA). Five microliters of extracted product was plated with 45 microliters of AgPath-ID One Step RT-PCR master mix (Applied Biosystems, Life Technologies, Carlsbad, CA, USA) containing the appropriate primers and probes at the manufacturer’s recommended concentration. Reactions were performed and analyzed on a 7500 real-time PCR system (Applied Biosystems).

RNA-Seq

A subset of EVD68-positive nasopharyngeal aspirates was selected from each year (n = 30) with an EVD68 prevalence >2% from 2009–2018. Selection was based upon lowest cycle threshold values (i.e., highest viral nucleic acid concentration), such that cost-effective next generation sequencing could be performed by batching (at most) a dozen specimens. Residual nasopharyngeal aspirate specimens were available from 4 cases of AFM in 2016 that tested positive for EVD68. All selected specimens were subjected to total RNA extraction via Qiamp viral RNA mini kit (Qiagen, Hilden, Germany) following the manufacturer’s instructions. This included on-column DNase treatment with RNase-free DNase set (Qiagen). The purified RNA

was immediately spin-vacuumed for 30 min to concentrate RNA for library preparation. Libraries were prepared with the TruSeq RNA kit (Illumina, San Diego, CA, USA) following the manufacturers recommendations. Libraries were pooled based upon estimated read counts needed for whole genome assembly. Pools were sequenced on a HiSeq 2500 (Illumina) to obtain roughly 20 million reads per sample. Two samples were randomly selected for repeat extraction, library preparation and sequencing to evaluate reproducibility of whole genome analysis.

As a quality control measure, 2 samples were randomly selected for repeat extraction, library preparation, sequencing and assembly. These 2 samples had 100% identity with our initial result across the entire polyprotein suggesting differences observed between samples are not the result of error (data not shown).

Bioinformatic Analysis

Sequencing fastq data was submitted to Sequence Read Archive (SRA) with the submission number: SUB4926426.

Non-redundant Selection of EVD68 Representative Genomes

As of December 6, 2018, there are 187 complete EVD68 genomes deposited in the NCBI RefSeq database. To de-replicate all the deposited genomes, similar genomes were clustered at 99% sequence identity using cd-hit (*3*) with the following non-default parameters: -c 0.99 -aS 0.95 -g 1 -d 0 -bak 1, represented as *evd68-nr99*. After de-replication, 55 non-redundant representative EVD68 genomes were used for downstream analysis.

EVD68 RNA-Seq Analysis

Raw sequencing data were processed using the shotgun metagenomics sequencing bioinformatics pipeline Sunbeam (*4*), including quality control, removing host sequences and masking low-complexity regions. To collect all the EVD68 reads from the sequencing data, quality-controlled reads were aligned to the evd68-nr99 dataset using the Burrows-Wheeler Aligner, version 0.7.17 (*5*). We then conducted de novo assembly on the collected EVD68 reads using either Spades version 3.12.0 (*6*) or MEGAHIT version 1.1.3 (*7*).

Phylogenetic Analysis

Polyprotein gene regions were identified by nucleotide alignment to EVD68 reference genomes using blastn. Within the resultant polyprotein regions, sequences for each protein (VP2, VP3, VP1, 2A, 2B, 2C, 3A, 3C, and 3D) were further annotated by alignment to protein

sequences from the reference genomes using blastp. For each protein, a multiple sequence alignment was constructed using MUSCLE (*8*). The multiple sequence alignments were concatenated, and a maximum-likelihood phylogenetic tree was inferred using RAxML (*9*) (model parameter set to PROTGAMMAGTR).

References

1. Kares S, Lönnrot M, Vuorinen P, Oikarinen S, Taurianen S, Hyöty H. Real-time PCR for rapid diagnosis of entero- and rhinovirus infections using LightCycler. J Clin Virol. 2004;29:99–104. PubMed http://dx.doi.org/10.1016/S1386-6532(03)00093-3

2. Wylie TN, Wylie KM, Buller RS, Cannella M, Storch GA. Development and evaluation of an enterovirus D68 real-time reverse transcriptase PCR assay. J Clin Microbiol. 2015;53:2641–7. PubMed http://dx.doi.org/10.1128/JCM.00923-15

3. Fu L, Niu B, Zhu Z, Wu S, Li W. CD-HIT: accelerated for clustering the next-generation sequencing data. Bioinformatics. 2012;28:3150–2. PubMed http://dx.doi.org/10.1093/bioinformatics/bts565

4. Clarke EL, Taylor LJ, Zhao C, Connell A, Lee JJ, Fett B, et al. Sunbeam: an extensible pipeline for analyzing metagenomic sequencing experiments. Microbiome. 2019;7:46. PubMed http://dx.doi.org/10.1186/s40168-019-0658-x

5. Li H, Durbin R. Fast and accurate short read alignment with Burrows-Wheeler transform. Bioinformatics. 2009;25:1754–60. PubMed http://dx.doi.org/10.1093/bioinformatics/btp324

6. Bankevich A, Nurk S, Antipov D, Gurevich AA, Dvorkin M, Kulikov AS, et al. SPAdes: a new genome assembly algorithm and its applications to single-cell sequencing. J Comput Biol. 2012;19:455–77. PubMed http://dx.doi.org/10.1089/cmb.2012.0021

7. Li D, Liu CM, Luo R, Sadakane K, Lam TW. MEGAHIT: an ultra-fast single-node solution for large and complex metagenomics assembly via succinct de Bruijn graph. Bioinformatics. 2015;31:1674–6. PubMed http://dx.doi.org/10.1093/bioinformatics/btv033

8. Edgar RC. MUSCLE: multiple sequence alignment with high accuracy and high throughput. Nucleic Acids Res. 2004;32:1792–7. PubMed http://dx.doi.org/10.1093/nar/gkh340

9. Stamatakis A. RAxML version 8: a tool for phylogenetic analysis and post-analysis of large phylogenies. Bioinformatics. 2014;30:1312–3. PubMed http://dx.doi.org/10.1093/bioinformatics/btu033

10. Wang G, Zhuge J, Huang W, Nolan SM, Gilrane VL, Yin C, et al. Enterovirus D68 Subclade B3 Strain Circulating and Causing an Outbreak in the United States in 2016. Sci Rep. 2017;7:1242. PubMed http://dx.doi.org/10.1038/s41598-017-01349-4

11. Greninger AL, Naccache SN, Messacar K, Clayton A, Yu G, Somasekar S, et al. A novel outbreak enterovirus D68 strain associated with acute flaccid myelitis cases in the USA (2012-14): a retrospective cohort study. Lancet Infect Dis. 2015;15:671–82. PubMed http://dx.doi.org/10.1016/S1473-3099(15)70093-9

12. Grantham R. Amino acid difference formula to help explain protein evolution. Science. 1974;185:862–4. PubMed http://dx.doi.org/10.1126/science.185.4154.862

Appendix Table 1. Amino acid substitutions in circulating EVD68*

EVD68 gene region	Amino acid position no.	1962 Fermon Strain	Amino acid identity per year				
			2009	2012	2014	2016	2018
VP4	18†	I	I/v	I/v	I	V	V/i
VP2	128	S	A	A	A	A	A
VP2	136†	R	K	K	K	R	R
VP2	142†	S	A/T	T	T	A	A
VP2	143†	N	G/e	G/e	G	T	T
VP2	204	H	Y/h	Y/h	H	H	H
VP2	207†	T	N/t	N/t	N	T	T
VP2	213	N	D/T	D/T	D	D	D
VP2	220†	R	E	E	E	A	A/t
VP2	236	I	L	L	L	L	L
VP2	264	V	A	A	A	A	A
VP2	280	V	I	I	I	I	I
VP2	291†§	M	M/V/t	T/v	T	M	M
VP2	294	V	M	M	M	M	M
VP3	341†§	V	V/a	V/a	A	V	V
VP3	357	I	V	V	V	V	V
VP3	363	M	V	V	V	V	V
VP3	364	I	V	V	V	V	V
VP3	376	D	E	E	E	E	E
VP3	379	N	V	V	V	V	V
VP3	391	Q	L	L	L	L	L
VP3	392	A	T	T	T	T	T
VP3	394	L	V	V	V	V	V
VP3	480†	I	V	V	V	I/v	I
VP3	497	S	N	N	N	N	N
VP1	553†	S	L	L/s	L	I/L	I
VP1	558†	G	A	A	A	G	G
VP1	565	V	I	I	I	I	I
VP1	628	G	S	S	S	S	S
VP1	642	N	N/d	D/n	D	D	D
VP1	644	A	A/t	T/a	T	T	T
VP1	647	S	E/a	A/e	A	A	T
VP1	649	G	Q	Q	Q	Q	Q
VP1	650	T	T/a	A/t	A	A/t	T
VP1	651	H	D	D	D	D	D
VP1	694	N	S	S	S	S	S
VP1	695	N	S	S	S	S	N/t
VP1	696	D	S/N	N	N	N	N
VP1	700	M	M/v/t	V/t	V	V	V
VP1	720	K	K/E	E	E	E	E
VP1	721	E	Q	Q	Q	Q	Q
VP1	746†	M	M	M	I	M/i	M
VP1	759	V	I/V	V	V	V	V

EVD68 gene region	Amino acid position no.	1962 Fermon Strain	Amino acid identity per year				
			2009	2012	2014	2016	2018
VP1	770†	N	S	S	N	T	T
VP1	795	V	I/v	V/i	V	V	V
VP1	821	M	L	L	L	L	L
VP1	832	K	R/k	K/r	K	K	K
VP1	835	D	E	E	E	E	E
VP1	836	T	R	R	R	R	R
VP1	840	T	A	A	A	A	A
VP1	842†	N	N/s	S/n	S	N	N
VP1	849	A	D	D	D	D	D
VP1	852	T	K	K	K	K	K
VP1	860†§	**T**	T/n	N/t	N	T	T
2A	883†	I	T/A	A/t	A	T	T
2A	898†	V	V	V	V	I	I
2A	927†§	**D**	D/n	N/d	N	D/n	D
2A	960	A	A/v	V/a	V	V	V
2C	1108†§	**S**	S/G	G/s	G	S	S
2C	1165	K	S	S/N	N	N	N
2C	1179	R	Q	Q	Q	Q	Q
2C	1205	S	A	A	A	A	A
2C	1209†	A	V/t	V/t	V	M	M
2C	1380	N	D/g	G/d	G	G/s	G
2C	1384†	T	T/I	T	T	A/v	V
3A	1450	A	T	T	T	T	T
3A	1483†						
3A	1490†	V	L	L	I	L	L
3C	1594	I	T	T	T	T	T
3C	1598†	N	N	N	N	D/n	D
3C	1603†	R	K/r	R/k	K	R/k	R
3C	1641	C	Y	Y	Y	Y	Y
3D	1742	M	V	V	V	V	V
3D	1772	S	S/p	P/s	P	P	P
3D	1826	N	S	S	S	S	S
3D	1832	E	D	D	D	D	D
3D	1866	S	T/s	S/t	S	S	S
3D	2005†§	R	R/k	K/r	K	R	R
3D	2008	V	I	I	I	I	I
3D	2009	I	V	V	V	V	V
3D	2038	K	R	R	R	R	R
3D	2076†	H	H/q	Q/h	Q	H	H
3D	2091†	I	V	V	I	V	V

*Yellow highlighting indicates 37 aa substitutions between the prototype Fermon strain and all circulating strains in Philadelphia during 2009–2018.
†2014 clade B1 and 2016 clade B3 viruses circulating in New York state described in (*10*).
§6 aa residues that were implicated for possible neurotropism because of conservation between other neurotropic enteroviruses with different identities from nonneurotropic enteroviruses as well as rhinoviruses described in (*11*).

Appendix Table 2. Nucleotide changes in the enterovirus D68 5′ untranslated region*

Numbering based on 1962 Fermon strain, nucleotide position no.	Fermon	Nucleotide identity per year				
		2009	2012	2014	2016	2018
29	Deletion	C	C	C	C	C
107	C	C	C	C	U	U
127	A	A/U	U/A	U	G	G
620	U	U	U	U	U	DEL
629	U	C/U	C/u	C	U/C	U
648	G	A	A	A/c	A	G
654	U	U	U	U	C	C
662	U	C/U	C	C	C	U
669	U	U/c	C/u	C	U	U
678	C	U	U	U	C	C
682–704	23 bp sequence	Deletion	Deletion	Deletion	Deletion	Deletion
722–733	11 bp sequence	4/7 Deletion	3/4 Deletion	Deletion	Deletion	Deletion

*Yellow highlighting indicates 37 aa substitutions between the prototype Fermon strain and all circulating strains in Philadelphia during 2009–2018.

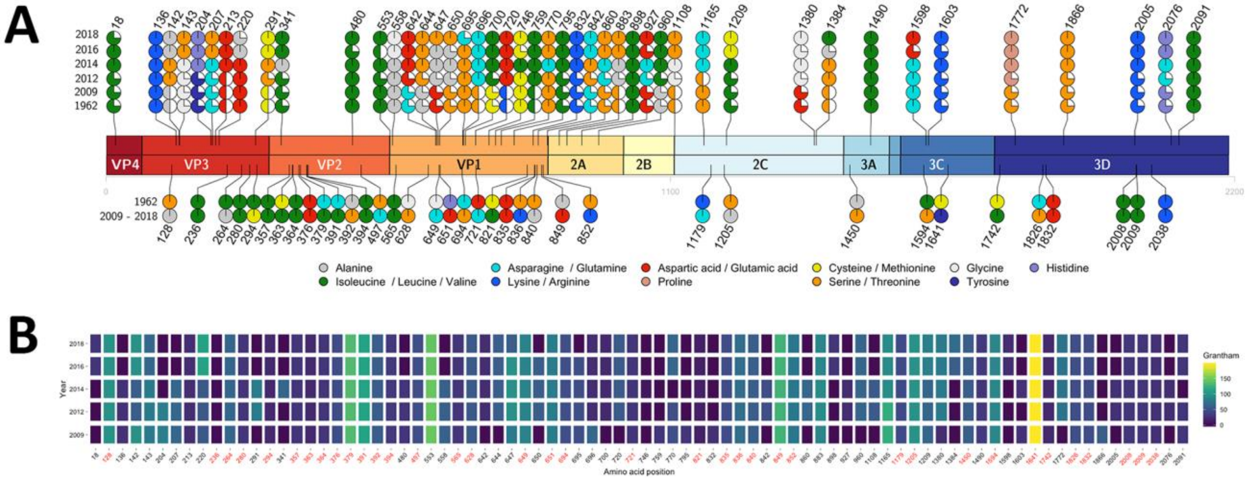

Appendix Figure. Amino acid changes in EVD68 circulating from 2009–2018. (A) Amino acid changes between years, from EVD68 whole genome sequences circulating in Philadelphia, are shown in the top plot. Amino acid changes between the 1962 Fermon strain and conserved between Philadelphia strains circulating from 2009–2018 are shown in the bottom plot. Amino acids are grouped by Shapely color scheme with corresponding colors indicated in the legend. White pie wedges indicate minor amino acid populations present (Appendix Table 1 for specific amino acids). (B) Grantham score (*12*) plot of amino acid changes among circulating Philadelphia strains compared to the 1962 Fermon strain. The x-axis indicates the amino acid position and the y-axis indicates the year between 2009–2018. The color legend indicates the distance score.
